# Effect of Adjusted Gas Nitriding Parameters on Microstructure and Wear Resistance of HVOF-Sprayed AISI 316L Coatings

**DOI:** 10.3390/ma12111760

**Published:** 2019-05-30

**Authors:** Pia Kutschmann, Thomas Lindner, Kristian Börner, Ulrich Reese, Thomas Lampke

**Affiliations:** 1 Materials and Surface Engineering Group, Institute of Materials Science and Engineering, Chemnitz University of Technology, D-09107 Chemnitz, Germany; th.lindner@mb.tu-chemnitz.de (T.Li.); thomas.lampke@mb.tu-chemnitz.de (T.La.); 2 Härterei Reese Chemnitz GmbH & Co. KG, 09117 Chemnitz, Germany; KBoerner@haerterei.com (K.B.); UReese@haerterei.com (U.R.)

**Keywords:** thermal spraying, high velocity oxy-fuel (HVOF), S-phase, expanded austenite, 316L, stainless steel, thermochemical treatment, hardening, gas nitriding

## Abstract

Gas nitriding is known as a convenient process to improve the wear resistance of steel components. A precipitation-free hardening by low-temperature processes is established to retain the good corrosion resistance of stainless steel. In cases of thermal spray coatings, the interstitial solvation is achieved without an additional surface activation step. The open porosity permits the penetration of the donator media and leads to a structural diffusion. An inhomogeneous diffusion enrichment occurs at the single spray particle edges within the coating’s microstructure. A decreasing diffusion depth is found with increasing surface distance. The present study investigates an adjusted process management for low-temperature gas nitriding of high velocity oxy-fuel-sprayed AISI 316L coatings. To maintain a homogeneous diffusion depth within the coating, a pressure modulation during the process is studied. Additionally, the use of cracked gas as donator is examined. The process management is designed without an additional surface activation step. Regardless of surface distance, microstructural investigations reveal a homogeneous diffusion depth by a reduced processing time. The constant hardening depth allows a reliable prediction of the coatings’ properties. An enhanced hardness and improved wear resistance is found in comparison with the as-sprayed coating condition.

## 1. Introduction

Thermochemical treatment is a common process for functionalizing the surface of stainless steels to improve their wear characteristics. Depending on the temperature-time regime during the process, the nitrogen enrichment leads to the formation of precipitates at temperatures above 500 °C, or an interstitial solid solution below 450 °C. Supersaturation of the austenitic matrix decisively expands the lattice parameters. This phase condition is named S-phase or expanded austenite [1,2,3].

Hardening of austenitic stainless steels comprises an initial surface activation step in order to remove the passive layer. Special equipment is required to ensure absence of repassivation during the diffusion enrichment. This significantly increases the complexity of the process management and often causes high costs. In contrast, classical thermochemical treatments possess clear economic benefits; in particular, the high flexibility and the relatively low processing costs are responsible for larger-scale industrial application of the gas nitriding process [4,5].

Different parameter settings related to bulk materials aim to increase process efficiency in order to reduce processing time or enhance hardness depth. Aleekseeva et al. applied a high-pressure gas nitriding process at a high temperature of 1150 °C, a high pressure of 150 MPa and a duration of 3 h. The diffusion layer in martensitic steels reaches 1.5–2 mm thickness [5]. In contrast, Wolowiec-Korecka et al. evaluated the low-pressure nitriding process for construction alloy steels and low-carbon non-alloy steel at a temperature of 560 °C, a pressure of 0.0026 MPa and a duration of up to 6 h. Both authors derived from their experiments an increase in the nitrogen concentration at the steel surface together with a high diffusion rate. For low-pressure nitriding process, these findings are explained by the surface phenomena adsorption, dissociation and desorption [6].

In the case of stainless-steel treatments, several process adjustments are required to maintain corrosion resistance. To prevent chromium depletion the process, temperature is subjected to a limitation. By using a low-temperature treatment, the diffusion depth is limited to a few microns, whereby high temperature treatment results in recrystallization. Furthermore, the passivation layer acts as a diffusion barrier, thus an activation step is necessary. This causes the risk of an inhomogeneous diffusion layer growth by incompleteness of surface activation [3]. 

In general, thermochemical treatments are applied to bulk materials. Nevertheless, several researchers have combined thermal spray processes and thermochemical treatment and proved the feasibility of stainless-steel coatings using various industrial surface hardening processes [4,7,8,9,10,11,12,13,14,15,16,17]. Plasma, molten metal and salt bath processes show comparable results to bulk material treatments of the same steel type [7,9,10,11,12,13,14,17]. Conversely, gas nitriding of thermal sprayed coatings, as described in [4,8,15,16], improves the diffusion depth of the enrichment media. Nestler and Lindner, who conducted a gas nitriding process with stainless steel high-velocity oxy-fuel (HVOF) coatings without a surface activation step, explained this finding by the characteristic open porosity of thermal sprayed coatings. The effect is shown for conventional treatment at temperatures above 500 °C, as well as low-temperature processes [4,8,15]. 

The present study focuses on the modification of a classical gas nitriding process for AISI 316L HVOF thermal-sprayed coatings. The modification comprises a gas pressure modulation and a controlled process gas regime. The gas pressure modulation intends to utilize the open porosity effect of thermal sprayed coatings to increase the nitrogen diffusion depth. An increase of the nitrogen supply at the coating surface is aimed with a controlled nitriding. The temperature-time regime of the gas nitriding process is kept constant at a temperature of 420 °C and a duration of 10 h. The nitriding depth, phase composition, hardness and the wear resistance of the sprayed coatings are compared considering the different nitriding process regimes.

## 2. Materials and Methods

The AISI 316L coatings were produced using an HVOF K2 system (GTV GmbH, Luckenbach, Germany) with the parameters given in Table 1. The coating was deposited on Ø 40 × 8 mm steel samples of the same grade. Prior to the coating’s deposition, the samples were grit-blasted with EK-F24 (Treibacher Industrie AG, Althofen, Austria), a pressure of 3 bar and a distance of 150 mm, under an angle of 70°, then ultrasonically cleaned for 5 min. The coating material was a gas-atomized powder with a particle size fraction of −53 + 20 µm (80.46.1, GTV GmbH, Luckenbach, Germany). After coating production, the samples were ground and polished up to mesh 1000 in order to examine the surface properties after nitriding and the wear tests. Thereby, the coating thickness averaged about 270 µm.

Gas nitriding was performed at 420 °C for 10 h in an industrial vacuum chamber retort heat-treatment furnace (WMU Wärmebehandlungsanlagen, Bönen, Germany) equipped with an ammonia cracker (O-SG-9/5, KGO GmbH, Wetter, Germany) and a hydrogen sensor (STANGE Elektronik GmbH, Gummersbach, Germany) to determine the nitriding potential. The temperature and duration were held constant for all trials. During the trials, the process regime was changed firstly with an industrial process (420 °C/10 h), secondly by adding a pressure modulation (420 °C/10 h PM) and thirdly by performing a pressure modulation in conjunction with a controlled process regime (420 °C/10 h PM + C, Table 2). Ammonia NH_3_ with a volume flow of 1000 l/h was used as process gas and mixed with dissociated ammonia in the controlled regime. The amount of cracked ammonia was adjusted according the predetermined nitriding potential maintaining the batch volume. The hydrogen amount was approximately five to six times higher in the controlled regime. The excess pressure in the chamber was varied between 2 mbar and a maximum operation pressure of 50 mbar in a cycle time as short as possible (<10 min). A surface activation step was not considered in the preparation of the gas nitrided samples.

The microstructural characterisation of the nitrided samples included the preparation of cross-sections by hot embedding, grinding and polishing. The cross-sections were wet-etched with the colour etchant Beraha II immediately after polishing. The etching duration varied between 10 and 15 s according to the colour change on the cross-sections. In contrast with the austenitic phase, the supersaturated matrix showed no colouring. Images were recorded using an optical microscope GX51 (Olympus, Shinjuku, Japan) equipped with a SC50 camera (Olympus, Shinjuku, Japan). The nano-indentation was applied at the etched cross-section using a UNAT nano-indenter (ASMEC GmbH, Radeberg, Germany) with a Berkovich tip. In order to determine the hardness values of the different phases, quasi-static measurements were performed with a load of 10 mN and at least 15 repetitions based on DIN EN ISO 14577-1 [18]. The solid solution of nitrogen in the face-centered cubic lattice was investigated by X-ray diffraction (XRD). A D8 DISCOVER diffractometer (Bruker AXS, Billerica, MA, USA) operating with Co Kα radiation (U: 40 kV; I: 40 mA) was used to measure in a diffraction angle (2θ) range from 20° to 130° with a step size of 0.01° and 1.5 s/step. Due to the use of a 1D Lynxeye XE detector (Bruker AXS, Billerica, MA, USA), this corresponded to 288 s/step.

Wear tests were conducted to verify the success of the thermochemical post-treatment. In comparison to the coating in the as-sprayed condition, the gas nitrided samples were tested in ball-on-disk and reciprocating ball-on-plane tests. The ball-on-disk test was carried out with Tetra Basalt Tester (Tetra GmbH, Ilmenau, Germany) based on ASTM G 99 [19] as a dry sliding system and the reciprocating ball-on-plane test was performed with a Wazau SVT 40 device (Wazau GmbH, Berlin, Germany) based on ASTM G 133 [20] as a dry couple. Parameters are given in Table 3. After the tribological testing, the wear tracks were evaluated with contact stylus instrument Hommel Etamic T8000 (Jenoptik GmbH, Villingen-Schwenningen, Germany) to determine the wear area after ball-on-disk testing. For the other test, wear volume was measured with an optical 3D profilometer MikroCAD (LMI Technologies Inc., Burnaby, Canada). The wear tracks were analysed using a scanning electron microscope (SEM) LEO 1455VP (Zeiss, Jena, Germany).

## 3. Results

### 3.1. Microstructural Analysis

**Figure 1:**
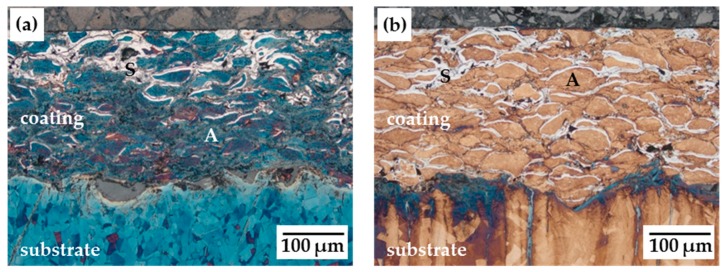
Cross-sectional micrographs of AISI 316L HVOF coating after gas nitriding at (**a**) 420 °C/10 h and (**b**) 420 °C/10 h PM. (S: S-phase, A: austenitic phase).

**Figure 2:**
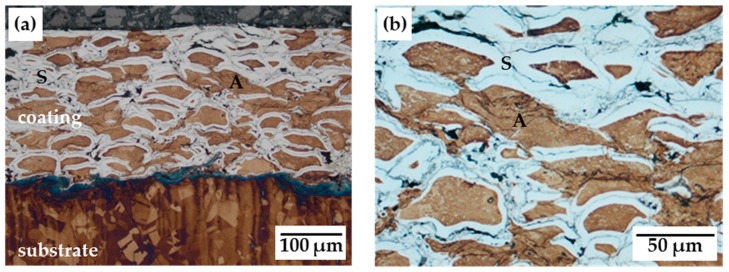
Cross-sectional micrographs of AISI 316L HVOF coating after gas nitriding at 420 °C/10 h with PM + C: (**a**) overview; (**b**) detailed view of the coating. (S: S-phase, A: austenitic phase.)

A low-temperature gas nitriding process at 420 °C for 10 h led to nitrogen enrichment in AISI 316L HVOF sprayed coatings without an initial activation step. Figure 1 and Figure 2 illustrate the nitrided coatings depending on the applied process regime. The white layer in the Beraha II-etched cross-sections refers to the S-phase (S), whereas the blue and brown areas represent the initial austenite phase (A). The different colouration of the austenite phase is the result of the etching duration and the preparation of the etchant agent applied for each sample.

The formation of the S-phase was inhomogeneous along the coating thickness, starting at the single spray particle’s edge. This confirms the gas permeability of the porous thermal spray coating’s microstructure. In the as-sprayed condition, the coatings exhibited a porosity of 1.6%. A detailed description of the coating’s microstructure before nitriding is given in [8]. The penetration depth was enhanced by a pressure modulation of the gas donator (Figure 1b). The gas exchange improved through the coating’s open porosity. In contrast, a plasma thermochemical treatment [7,9,10,12,13,14] or precipitation hardening [4,8] of thermal spray steel coatings generated a homogenous S-phase or compound layer up to 20 µm or above 100 µm in depth, respectively.

Significant improvements can be achieved by using dissociated ammonia for the same process duration. The diffusion zone increased at the spray particle’s edge and a uniform diffusion depth was reached up to the substrate surface (Figure 2a). Small particles were nitrided completely (Figure 2b). The coatings exhibited a similar S-phase fraction in comparison with results for a 30-h duration without a controlled gas regime [8]. Hence, a notable time reduction of the process was realised. Consequently, the use of dissociated ammonia under modulated gas pressure ensured a high nitrogen supply within the coating’s structure. As a result, the diffusion in depth and a homogenous distribution of the austenite and S-phase were improved. These results are in accordance with effects recognized for high- and low-pressure gas nitriding of bulk materials under similar process conditions [5,6].

**Figure 3:**
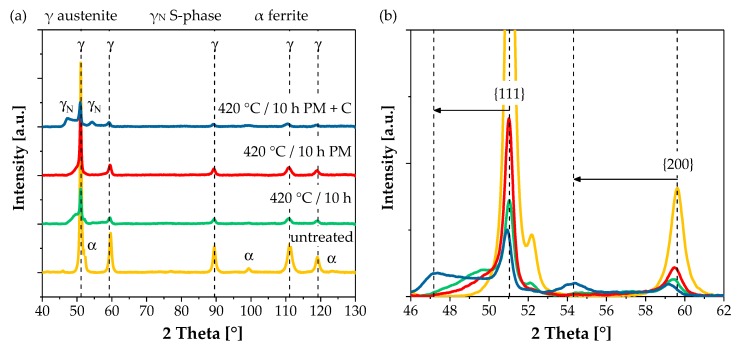
XRD diagrams of AISI 316L HVOF coatings before and after gas nitriding: (**a**) overview; (**b**) detailed view of the peak shift of {111} and {200} lattice planes.

Figure 3 shows the XRD patterns of the HVOF coatings considering the different treatment states in conjunction with the as-sprayed condition. The untreated coating exhibited characteristic peaks of the austenite phase and additional minor peaks that corresponded to the ferrite phase. Depending on the setting of the gas nitriding process, the peak intensity decreased. Additional peaks of the expanded austenite appeared at lower angles compared to the initial austenitic phase. The peaks shifted and intensity increased with the amount of the S-phase fraction. A higher magnification of the peak shift for the lattice planes {111} and {200} is illustrated in Figure 3b. The broad S-phase peak indicates a superposition of different lattice expansions and an inhomogeneous interstitial dissolution of nitrogen within the coating’s microstructure. Higher lattice expansion equates a higher nitrogen enrichment, as observed for the pressure-modulated and controlled gas nitriding. The results differ from the XRD pattern of a low-temperature thermochemical-treated bulk material and a plasma thermochemical-treated thermal spray AISI 316L coating. These revealed clear peak shifts of the austenite lattice planes, indicating the S-phase [2,7,15]. Nitride phases like CrN or Fe_4_N can be excluded by the XRD measurements.

### 3.2. Hardness and Wear Resistance

The coating’s cross-sections showed a two-phase microstructure after gas nitriding. Table 4 summarizes the nano-hardness of the different microstructure domains. The austenitic phase of the nitrided samples exhibited a nano-hardness of 440 HV_10mN_ on average. In comparison with the untreated state, the increase in hardness was affected by the nearby S-phase. The S-phase was more than twice as hard and ranged between 870 and 1000 HV_10mN_. The hardness values are in accordance with the results of plasma nitrided cold sprayed AISI 316L coatings [13]. In general, a strong shift of the diffraction peak corresponded to high nano-hardness values. This relationship was in particular valid for greater portion of S-phase fractions. All samples showed a superposition of the dissolved nitrogen lattice expansion. A decreasing nitrogen concentration from the enriched spray particles’ edges was reasonable for a broad peak appearance. Hence, a gradation in hardness can be assumed.

**Figure 4:**
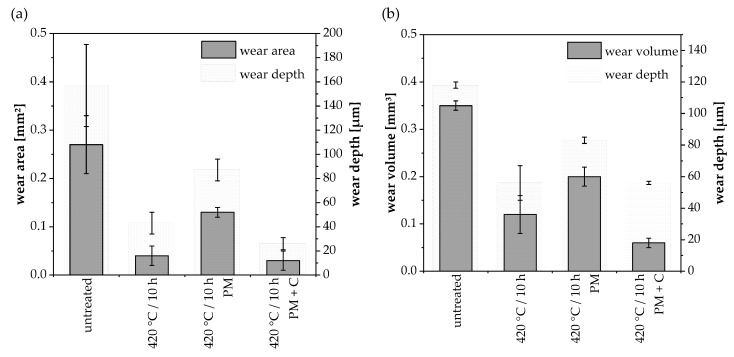
Results of the wear tests of AISI 316L coatings before and after gas nitriding, (**a**) ball-on-disk test and (**b**) reciprocating ball-on-plane test.

**Figure 5:**
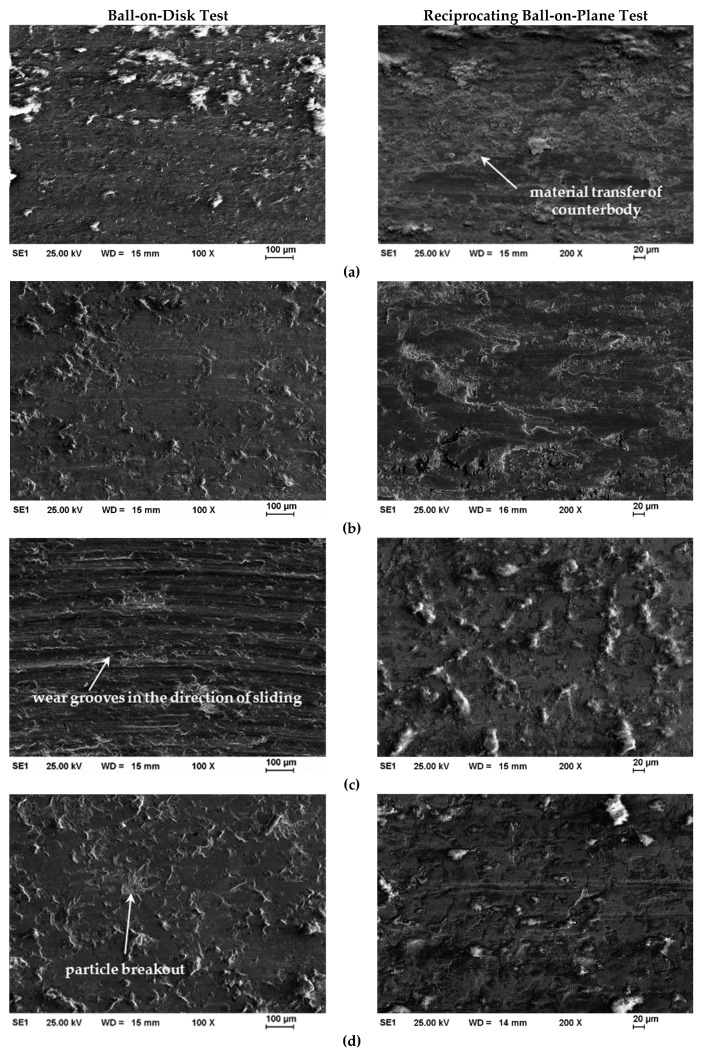
SEM micrographs of the wear tracks of AISI 316L coatings before and after gas nitriding after the ball-on-disk and reciprocating ball-on-plane tests. (**a**) AISI 316L HVOF coating in untreated condition; (**b**) AISI 316L HVOF coatings after gas nitriding at 420 °C/10 h; (**c**) AISI 316L HVOF coatings after gas nitriding at 420 °C/10 h PM; (**d**) AISI 316L HVOF coatings after gas nitriding at 420 °C/10 h PM + C.

Low-temperature gas nitriding improved the wear resistance of the coatings in the ball-on-disk and reciprocating ball-on-plane test conditions (Figure 4). The wear rate was deeply influenced by the wear mechanism. Because of the high deformation of the austenite phase, the untreated AISI 316L steel coating showed severe wear in both wear tests (Figure 5a). Adhesive wear prevailed also in the gas nitrided samples at 420 °C/10 h, which showed a significant decrease in wear area and volume, respectively (Figure 5b). In addition, particle breakouts increased in the reciprocating ball-on-plane test due to the frequent contact of the surface with the counterbody. In comparison with this, the wear of the pressure-modulated sample increased. The higher nano-hardness values resulted in a certain change in wear mechanism. Figure 5c shows a predominately abrasive wear with deep grooves in the direction of sliding for the ball-on-disk test. These were caused by the breakout of hardened spray particles acting as abrasives in combination with inhomogeneous nitriding at the surface (Figure 1b). However, the adhesive wear dominated in the reciprocated ball-on-plane test.

The highest wear resistance was proven for the gas nitrided samples with dissociated ammonia in the pressure-modulated regime (Figure 4) possessing a homogenous nitrogen enrichment and S-phase fraction. The main wear mechanism can be assigned to adhesive wear indicated by plastic deformation and minor particle breakouts in both tests (Figure 5d). A reduction of approximately 89% in wear area for the ball-on-disk test condition and 83% in wear volume for the reciprocated ball-on-plane test condition can be achieved by thermochemical treatment of coatings. In conclusion, a post gas nitriding of AISI 316L HVOF coatings led to a significant wear improvement with a general better performance in the ball-on-disk test.

## 4. Conclusions

The microstructural evolution in HVOF-sprayed AISI 316L coatings during gas nitriding was studied with respect to parameter settings. A pressure modulation within the post-treatment step improved the penetration depth. It was found that the diffusion depth was decisively increased by using dissociated ammonia. In addition to this, the process efficiency related to the processing time was improved. A homogeneous diffusion layer growth within the single spray particles from the coatings’ surfaces to the substrate was proven for an adjusted gas donator composition and a pressure-modulated process management. This can be explained by a higher activity and constant exchange with renewing of the donator media. The expanded austenite showed significantly increased hardness in comparison with the initial austenitic phase. Gas nitriding of AISI 316L HVOF coatings resulted in a significant enhanced wear resistance. Due to the thermochemical treatment, a change in wear mechanism could be recognized. Abrasive wear was indicated by grooves within the austenitic phase. These were caused by worn out particles of the hard S-phase. In the case of a homogenous hardened surface, the adhesive wear occurred, resulting in best wear resistance shown by a combined pressure-modulated and controlled gas nitriding process. From the results of the present study, the general feasibility of the novel processing approach is confirmed. The constant hardening depth within the single spray particles allows a reliable prediction of the coating properties regardless surface distance.

## Figures and Tables

**Table 1:** Setting parameter of AISI 316L coatings for the HVOF K2 system.

Kerosene	Oxygen	λ	Nozzle	Powder Feed Rate	Carrier Gas	Spray Distance	Step Size	Surface Velocity
[l/h]	[l/min]		[mm/mm]	[g/min]	[l/min]	[mm]	[mm]	[m/s]
24	900	1.1	150/14	70	2 × 8	350	5	1

**Table 2:** Parameter adjustments during gas nitriding trials.

Trial	Process Regime	Pressure Modulation (PM) Over Pressure/Cycle Time	Controlled Process (C)
1	420 °C/10 h	2 mbar	NH_3_
2	420 °C/10 h PM	2–50 mbar/<10 min	NH_3_
3	420 °C/10 h PM + C	2–50 mbar/<10 min	NH_3_ + N_2_ + H_2_

**Table 3:** Setting parameters for the wear tests.

Ball-on-Disk Test		Reciprocating Ball-on-Plane Test	
Normal load [N]	20	Normal load [N]	26
Radius [mm]	5	Frequency [Hz]	40
Speed [rpm]	96	Time [s]	900
Cycles	15,916	Amplitude [mm]	0.5
Ø Al_2_O_3_ [mm]	6	Ø Al_2_O_3_ [mm]	10

**Table 4:** Hardness of the AISI 316L HVOF coatings before and after gas nitriding.

State of Treatment	Untreated	420 °C/10 h	420 °C/10 h PM	420 °C/10 h PM + C
Austenitic phase HV_10mN_	316 ± 59	462 ± 77	420 ± 39	449 ± 41
S-phase HV_10mN_		874 ± 136	1005 ± 65	971 ± 68

## References

[1] Christiansen T.L., Somers M.A.J. (2009). Low-temperature gaseous surface hardening of stainless steel: The current status. Int. Mater. Res..

[2] Bell T. (2008). Current status of supersaturated surface engineering S-phase materials. Key Eng. Mater..

[3] Bottoli F., Jellesen M.S., Christiansen T.L., Winther G., Somers M.A.J. (2018). High temperature solution-nitriding and low-temperature nitriding of AISI 316: Effect on pitting potential and crevice corrosion performance. Appl. Surf. Sci..

[4] Nestler M.C., Spies H., Hermann K. (1996). Production of duplex coatings by thermal spraying and nitriding. Surf. Eng..

[5] Alekseeva M.S., Gress M.A., Scherbakov S.P., Gerasimov S.A., Kuksenova L.I. (2017). The influence of high-pressure gas nitriding on the properties of martensitic steels. Met. Sci. Heat Treat..

[6] Wolowiec-Korecka E., Michalski J., Kucharska B. (2018). Kinetic aspects of low-pressure nitriding process. Vacuum.

[7] Adachi S., Ueda N. (2018). Wear and corrosions properties of cold-sprayed AISI 316L coatings treated by combined plasma carburizing and nitriding at low temperature. Coatings.

[8] Lindner T., Kutschmann P., Löbel M., Lampke T. (2018). Hardening of HVOF-sprayed austenitic stainless-steel coatings by gas nitriding. Coatings.

[9] Adachi S., Ueda N. (2014). Combined plasma carburizing and nitriding of sprayed AISI 316L coating for improved wear resistance. Surf. Coat. Technol..

[10] Park G., Bae G., Moon K., Lee C. (2013). Effect of plasma nitriding and nitrocarburizing on HVOF-sprayed stainless steel coatings. J. Therm. Spray Technol..

[11] Wielage B., Rupprecht C., Lindner T., Hunger R. Surface modification of austenitic thermal spray coatings by low-temperature carburization. Proceedings of the International Thermal Spray Conference & Exposition.

[12] Adachi S., Ueda N. (2012). Formation of S-phase layer on plasma sprayed AISI 316L stainless steel coating by plasma nitriding at low temperature. Thin Solid Films.

[13] Adachi S., Ueda N. (2015). Formation of expanded austenite on a cold-sprayed AISI 316L coating by low-temperature plasma nitriding. J. Therm. Spray Technol..

[14] Lindner T., Löbel M., Lampke T. (2018). Phase Stability and Microstructure Evolution of Solution-Hardened 316L Powder Feedstock for Thermal Spraying. Metals.

[15] Lindner T., Mehner T., Lampke T. (2016). Surface modification of austenitic thermal-spray coatings by low-temperature nitrocarburizing. IOP Conf. Ser. Mater. Sci. Eng..

[16] Piao Z.-Y., Xu B.S., Wang H.D., Wen D.H. (2013). Influence of surface nitriding treatment on rolling contact behavior of Fe-based plasma sprayed coating. Appl. Surf. Sci..

[17] Mindivan H. (2018). Investigating tribological charateristics of HVOF sprayed AISI 316 stainless steel coating by pulsed plasma nitriding. IOP Conf. Ser. Mater. Sci. Eng..

[18] ISO (2018). DIN EN ISO 14577-1: Metallic Materials-Instrumented Indentation Test for Hardness and Materials Parameters—Part 1: Test Method (ISO 14577-1:2015).

[19] ASTM International (2016). ASTM G 99 Standard Test Method for Wear Testing with a Pin-on-Disk Apparatus.

[20] ASTM International (2016). ASTM G 133 Standard Test Method for Linearly Reciprocating Ball-on-Flat Sliding Wear.

